# A Novel Variational Bayesian Method Based on Student’s *t* Noise for Underwater Localization

**DOI:** 10.3390/s25113291

**Published:** 2025-05-23

**Authors:** Haoqian Huang, Yutong Zhang, Chenhui Dong

**Affiliations:** College of Artificial Intelligence and Automation, Hohai University, Changzhou 213200, China; yutongz@hhu.edu.cn (Y.Z.); chenhui_dong@hhu.edu.cn (C.D.)

**Keywords:** student’s *t*-distribution, variational Bayesian, sliding window, multiple fading factors, multi-sensor fusion

## Abstract

In underwater environments, the presence of multipath effects can cause measurement outliers in acoustic sensors, leading to reduced estimation accuracy for integrated navigation. To address this issue, this paper proposes a sliding window variational Kalman filter based on Student’s *t*-distribution (SWVKF-ST) to improve state estimation accuracy. First, this method makes use of Student’s *t*-distribution to model heavy-tailed noise and adopts the inverse Wishart distribution as the prior for noise covariance, thereby enhancing robustness against heavy-tailed distributions. On this basis, the state variables and measurements within the sliding window are jointly estimated using the variational Bayesian framework, which helps mitigate the impact of unknown noise characteristics on state estimation. In addition, this method constructs multiple fading factors to prevent the degradation of estimation accuracy caused by excessive adjustment of the predicted error covariance matrix. Finally, the simulations and actual experiment validate that the SWVKF-ST outperforms the compared filters, achieving higher filtering precision and stronger robustness to outliers. The method effectively reduces the uncertainty in the measurement noise covariance matrix and demonstrates excellent adaptability in complex underwater environments.

## 1. Introduction

The ocean contains abundant biological resources and marine energy, making exploration and development a research hotspot worldwide. Autonomous underwater vehicles (AUVs) have been widely used in ocean exploration [1,2]. Accurate navigation information plays a critical role in ensuring the successful completion of AUV missions. Due to the influence of noise in the underwater environment, achieving high-precision underwater navigation remains a challenge. Inertial navigation system (INS) has the advantages of good autonomy and high concealment, but the errors of INS inevitably increase with time. At present, the commonly used underwater positioning technologies are based on acoustic wave propagation, including Doppler velocity log (DVL) and underwater acoustic positioning system. The use of DVL data can enhance the positioning accuracy of the INS. [3]. The ultra-short baseline (USBL) is one of the most common acoustic positioning systems used due to its easy placement and low price. The USBL obtains the relative position relationship between the acoustic array and the underwater transponder by measuring directions and range information between them [4]. Because individual sensors are prone to interference and performance degradation in underwater environments, the integrated INS/DVL/USBL navigation system plays a critical role in enhancing system stability and positioning accuracy. Therefore, the INS/DVL/USBL system has been widely used in the field of underwater vehicles [5]. In such challenging scenarios, multi-sensor information fusion is essential for achieving high-precision navigation [6,7].

Due to the widespread presence of noise and outliers in underwater environments, sensor data exhibit significant dynamic characteristics, further highlighting the urgent need for real-time and efficient updates of state information [8,9,10]. Therefore, filtering-based fusion localization methods are widely recognized for their strong real-time performance and high accuracy [11,12]. Traditional filter-based fusion localization methods, such as extended Kalman filter (EKF) and cubature Kalman filter (CKF), are based on the assumption that noise follows a Gaussian distribution [13]. However, in complex underwater environments, due to the inherent uncertainty of underwater noise, the difference between actual noise and predefined noise models can reduce the performance and accuracy of traditional multi-sensor data fusion algorithms.

To address the uncertainties associated with noise covariance matrices (NCMs), a variety of adaptive Kalman filter (AKF) methods have been developed. Notable examples include the Sage-Husa adaptive filter [14], multi-model approaches [15], and variational Bayesian adaptive Kalman filters (VBAKFs) [16]. Among these, VB has gained significant attention due to its robust capability to estimate noise parameters and quantify associated uncertainties using advanced mathematical frameworks. By utilizing Bayesian principles and selecting appropriate conjugate prior distributions, VB enables the estimation of measurement noise covariance matrices (MNCMs) and process noise covariance matrices (PNCMs), which has inspired the development of several improved algorithms [17,18]. In addition, AKF methods are not suitable for heavy-tailed non-Gaussian noise and are not robust to outlier measurements.

To address the issue of outliers, a robust Kalman filtering method called the statistical similarity measurement-based robust Kalman filter (SSMKF) was introduced. This method computes the posterior PDF of the state by maximizing a defined statistical similarity measure, thereby maximizing the lower bound of statistical similarity between the state vector and the predicted state vector [19]. However, SSMKF assumes that the nominal covariance of the noise corrupted by outliers is known. In practice, accurately determining noise parameters is challenging, which may lead to suboptimal performance of SSMKF in engineering applications. Therefore, a robust Student’s *t* Kalman filter (RSTKF) was proposed, employing a hierarchical state-space model based on Gaussian distributions. This method utilizes VB to jointly estimate the scale matrix, state vector, and degrees-of-freedom parameter, effectively enhancing estimation accuracy in the presence of outlier-contaminated noise [17]. To improve adaptability to noise covariance variations, Huang et al. [20] extended this concept by developing the sliding window variational adaptive Kalman filter (SWVAKF), which demonstrated adaptive learning capabilities for unknown PNCM and MNCM while maintaining high computational efficiency. Building upon this framework, the sliding window robust Kalman filter (SWRKF) was introduced, leveraging measurement data within a sliding window to effectively distinguish between model state variables and measurement anomalies. By adaptively adjusting the noise covariance, SWRKF achieves higher estimation accuracy. However, this improvement comes at the cost of increased computational complexity [21]. To enhance the tracking capability of the predicted error covariance matrix (PECM), Pan et al. [22] proposed a multi-fading factor-based strong tracking variational Bayesian adaptive Kalman filter (MST-VBAKF), incorporating unknown MNCM into the system state estimation. However, its estimation accuracy remains to be further improved.

**Figure 1:**
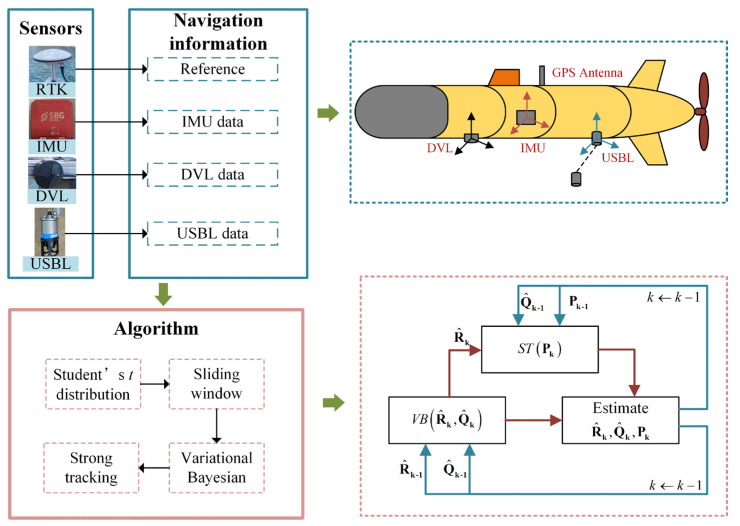
The structure of the proposed method.

Motivated by the need for improved accuracy and robustness in complex underwater environments, this paper proposes a novel Student’s *t*-based strong tracking sliding window variational Kalman filter (SWVKF-ST). The complete implementation flow is depicted in Figure 1. The proposed algorithm first acquires multiple state variables and measurements within a sliding window. It then employs variational Bayesian methods to obtain the optimal parameters of Student’s *t*-distribution for the PNCM and MNCM. Subsequently, the PECM is adaptively adjusted to mitigate the impact of outliers. The proposed method employs a sliding window to utilize these measurements over a period of time, thereby enhancing outlier detection capability and improving posterior accuracy. By using Student’s *t*-distribution for noise modeling, the adverse effects of heavy-tailed noise and outliers have been effectively alleviated. In addition, multiple fading factors based on the strong tracking principle prevent inappropriate adjustments of the PECM, thereby improving the stability of the algorithm. It has been demonstrated through simulation and experimental results that the proposed method achieves higher estimation accuracy and reliability than the compared methods in challenging underwater scenarios.

The main contributions of this paper can be summarized as follows:

- To address the heavy-tailed noise problem caused by outlier interference, the SWVKF-ST is proposed. Student’s *t*-distribution is adopted to model the noise, and auxiliary random variables are introduced to approximate it as a Gaussian hierarchical distribution.
- The proposed SWVKF-ST algorithm achieves further adaptive parameter updates through smoothing estimation within a sliding window. Through VB iteration, the state, measurement noise covariance, and auxiliary random variables are jointly estimated online, effectively mitigating the influence of outliers and improving estimation accuracy.
- To correct the predicted state covariance, the proposed SWVKF-ST algorithm constructs multiple fading factors. The optimal estimation of the measurement noise covariance is embedded as a time-varying parameter into the suboptimal fading factor based on the strong tracking principle, thereby improving the correction accuracy of the predicted state covariance.

The structure of this paper is organized as follows. Section 2 provides a comprehensive formulation of the problem under investigation. Section 3 introduces the proposed SWVKF-ST algorithm, detailing the construction of multiple fading factors, the selection of an appropriate noise model, and the variational approximation methodology. In Section 4, the effectiveness of the proposed algorithm is validated through extensive simulations and lake trial experiments, accompanied by a rigorous comparative analysis with other compared algorithms. Finally, Section 5 concludes the paper by summarizing the key findings and offering insights for future research directions.

## 2. Problem Formulation

The state-space model of a discrete linear stochastic system is defined as follows [23]:

(1) $$\left\{\begin{array}{l} x_{k}=F_{k}x_{k-1}+w_{k}\\ z_{k}=H_{k}x_{k}+v_{k} \end{array}\right.$$

where $x_{k}\in {\mathbb{R}}^{n}$ is the state vector; $z_{k}\in {\mathbb{R}}^{m}$ is the measurement vector; $F_{k}$ and $H_{k}$ are the state transition and observation matrices; $w_{k}$ and $v_{k}$ are, respectively, the state and measurement noises, both assumed to follow a Gaussian distribution.

Prediction and update are the two processes in the KF recursion:

(2) $${\hat{x}}_{k|k-1}=F_{k-1}{\hat{x}}_{k-1|k-1}$$

(3) $$P_{k|k-1}=F_{k-1}P_{k-1|k-1}F_{k-1}^{T}+Q_{k-1}$$

(4) $$K_{k}=P_{k|k-1}H_{k}^{T}{\left(H_{k}P_{k|k-1}H_{k}^{T}+R_{k}\right)}^{-1}$$

(5) $${\hat{x}}_{k|k}={\hat{x}}_{k|k-1}+K_{k}\left(z_{k}-H_{k}{\hat{x}}_{k|k-1}\right)$$

(6) $$P_{k|k}=\left(I-K_{k}H_{k}\right)P_{k|k-1}$$

where ${\hat{x}}_{k|k-1}$ and $P_{k|k-1}$ denote the predicted state vector and PECM and $K_{k}$ denotes the filtering gain matrix, respectively.

The traditional backward Rauch–Tung–Striebel (RTS) smoother which is known as KS involves the following steps (for k=T:1) [24]:

(7) $$P_{k|k-1}=F_{k}P_{k-1|k-1}F_{k}^{T}+Q_{k}$$

(8) $$G_{k-1}=P_{k-1|k-1}F_{k}^{T}P_{k|k-1}^{-1}$$

(9) $${\hat{x}}_{j-1|k}={\hat{x}}_{j-1|j-1}+G_{j-1}\left({\hat{x}}_{j|k}-F_{j}{\hat{x}}_{j-1|j-1}\right)$$

(10) $$P_{j-1|k}=P_{j-1|j-1}+G_{j-1}\left(P_{j|k}-P_{j|j-1}\right)G_{j-1}^{T}$$

where T is the sampling end point; $G_{k-1}$ is reverse smoothing gain. The forward recursion process follows the same procedure as the KF, while backward recursion helps to further mitigate fluctuations in the estimation results.

In Kalman filtering (KF), the process noise and measurement noise of the system are typically assumed to be zero mean Gaussian white noise. Under this assumption, KF can estimate the system state based on the minimum variance criterion and effectively fuse multi-sensor information using the information fusion principle and the covariance upper bound method. However, in underwater navigation scenarios, complex environmental disturbances such as acoustic reflections from irregular terrain and sudden changes in ocean currents often cause the noise to deviate from the ideal Gaussian assumption. The Gaussian noise model, which is light-tailed, is sensitive to outliers and thus struggles to handle the abrupt commonly found in underwater environments. In practice, underwater noise often exhibits significant heavy-tailed characteristics. In contrast, Student’s *t*-distribution, with its heavier tails and stronger robustness to outliers, can more accurately capture the statistical properties of such complex noise, thereby improving the stability and reliability of state estimation in the presence of extreme deviations. In such cases, modeling errors in the KF due to incorrect noise assumptions can significantly degrade estimation performance and even lead to filter divergence. Furthermore, KF relies on prior knowledge of the PNCM and MNCM, which is often difficult to obtain in dynamic environments.

**Figure 2:**
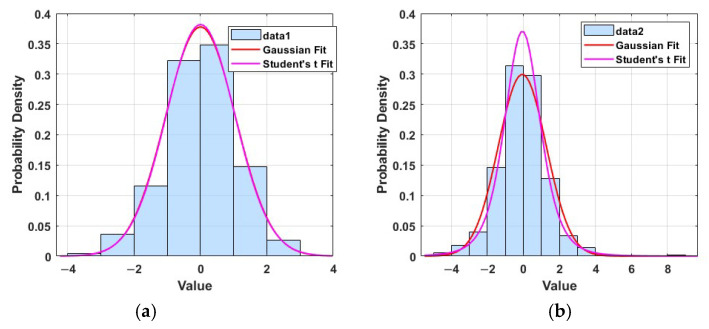
Comparison of Gaussian and Student’s *t*-distribution fitting under normal and heavy-tailed noise. (**a**) No heavy-tailed noise. (**b**) Heavy-tailed noise.

To validate the sensitivity of the Gaussian distribution to outliers, representative outliers are added to standard Gaussian-distributed samples for fitting analysis. Figure 2a shows that in the absence of heavy-tailed interference, both Gaussian and Student’s *t*-distributions demonstrate strong consistency with the data. In contrast, Figure 2b shows that after introducing heavy-tailed noise, the fitting ability of the Gaussian distribution decreases significantly, while Student’s *t*-distribution still effectively captures the tail characteristics of the data. This suggests that Student’s *t*-distribution exhibits enhanced robustness in the presence of outliers and heavy-tailed noise, making it more suitable for modeling non-Gaussian disturbances in sensor noise. Therefore, this paper adopts a modeling approach based on Student’s *t*-distribution to characterize the heavy-tailed noise present in sensor data, thereby enhancing the robustness and accuracy of the fusion algorithm in integrated navigation system.

## 3. Proposed Method

In this section, a novel strong tracking sliding window variational Kalman filter method based on Student’s *t*-distribution is proposed. The algorithm first collects multiple state variables and measurements within a sliding window, then applies variational Bayesian methods to optimize Student’s *t*-distribution parameters for the PNCM and MNCM. By using Student’s *t*-distribution for noise modeling, the impact of heavy-tailed noise and outliers is effectively reduced. Additionally, multiple fading factors based on strong tracking ensure appropriate PECM adjustments, enhancing algorithm stability.

### 3.1. Noise Modeling

To reduce the influence of measurement outliers on estimation accuracy, heavy-tailed noise is modeled with Student’s *t*-distribution. Assuming that the statistical properties of the noise change slowly, Student’s *t*-distribution of the state noise and measurement noise within the sliding window $\left[k-L+1,k\right]$ are approximated. The state transition probability density function (PDF) and the measurement likelihood PDF at time $j\in \left[k-L+1,k\right]$ are expressed as follows:

(11) $$p\left(x_{j}|x_{j-1},Q_{k}\right)=\mathrm{St}\left(x_{j};F_{j}x_{j-1},Q_{k},\omega \right)$$

(12) $$p\left(z_{j}|x_{j-1},R_{k}\right)=\mathrm{St}\left(z_{j};H_{j}x_{j-1},R_{k},\nu \right)$$

Student’s *t*-distribution can be approximated using a finite Gaussian hierarchical model. Therefore, the state transition PDF and the likelihood PDF are expressed as

(13) $$p\left(x_{j}|x_{j-1}\right)=\int N\left(x_{j};F_{j}x_{j-1},Q_{k}/\xi_{j}\right)G\left(\xi_{j};\omega /2;\omega /2\right)d\xi_{j}$$

(14) $$p\left(z_{j}|x_{j}\right)=\int N\left(z_{j};H_{j}x_{j},R_{k}/\lambda_{j}\right)G\left(\lambda_{j};\nu /2;\nu /2\right)d\lambda_{j}$$

where $G\left(\cdot ;\alpha ,\beta \right)$ denotes the Gamma PDF, in which α is shape parameter and β is rate parameter; $\xi_{j}$ and $\lambda_{j}$ are auxiliary random variables. Due to the non-closure of Bayesian recursive updates for Student’s *t*-distribution, an auxiliary random variable is introduced to express Student’s *t*-distribution as the Gaussian hierarchical PDF.

In Bayesian statistics, the inverse Wishart distribution is typically used as the conjugate prior for a Gaussian covariance matrix. Therefore, the inverse Wishart distribution can be used to represent the prior distribution of the covariance matrix $Q_{k}$ and $R_{k}$ to ensure conjugate inference:

(15) $$p\left(Q_{k}|z_{1:k-L}\right)=\mathrm{IW}\left(Q_{k};{\hat{t}}_{k|k-L},{\hat{T}}_{k|k-L}\right)$$

(16) $$p\left(R_{k}|z_{1:k-L}\right)=\mathrm{IW}\left(R_{k};{\hat{u}}_{k|k-L},{\hat{U}}_{k|k-L}\right)$$

where $\mathrm{IW}\left(\cdot ;\omega ,P\right)$ represents the distributions of the inverse Wishart with the degree-of-freedom (DOF) parameter ω and inverse-scale matrix P; ${\hat{t}}_{\left.k\right|k-L},{\hat{T}}_{\left.k\right|k-L},{\hat{u}}_{\left.k\right|k-L},{\hat{U}}_{\left.k\right|k-L}$ denote the corresponding DOF parameters and inverse-scale matrices. Since the PNCM and MNCM exhibit dynamic trends in practice, the posterior parameters can be propagated to the prior parameters through a forgetting factor ρ as shown below:

(17) $${\hat{t}}_{k|k-L}=\rho^{L}{\hat{t}}_{k-L|k-L},{\hat{T}}_{k|k-L}=\rho^{L}{\hat{T}}_{k-L|k-L}$$

(18) $${\hat{u}}_{k|k-L}=\rho^{L}{\hat{u}}_{k-L|k-L},{\hat{U}}_{k|k-L}=\rho^{L}{\hat{U}}_{k-L|k-L}$$

where ${\hat{t}}_{k-L|k-L},{\hat{T}}_{k-L|k-L},{\hat{u}}_{k-L|k-L},{\hat{U}}_{k-L|k-L}$ are the posterior DOFs and scale matrices of $Q_{k-L}$ and $R_{k-L}$; $\rho \in \left[0.9,1\right)$ is the forgetting factor, which reflects changes in PNCM and MNCM while introducing additional uncertainty.

### 3.2. Variational Approximations

The variational Bayesian method is an approximation technique that expresses complex posterior distributions using numerous prior distributions. For brevity, we define the set $\Psi_{k}≜\left\{x_{k-L},Q_{k},R_{k},\xi_{k-L+1},\lambda_{k-L+1}\right\}$. In the variational approximation framework, the variables to be estimated are assumed to be mutually independent. Consequently, the approximate PDF of the posterior joint PDF can be expressed as a product of free-form factors as

(19) $$p\left(\Psi_{k}|z_{1:k}\right)\approx q\left(x_{k-L:k}\right)q\left(Q_{k}\right)q\left(R_{k}\right)q\left(\xi_{k-L+1}\right)q\left(\lambda_{k-L+1}\right)$$

where $q\left(\cdot \right)$ is the PDF of the approximate posterior distribution.

The minimum Kullback–Leibler (KL) divergence between the approximate posterior PDF and its free-form factors is expressed as follows:

(20) $$\begin{array}{l} \left\{q\left(x_{k-L:k}\right)q\left(Q_{k}\right)q\left(R_{k}\right)q\left(\xi_{k-L+1}\right)q\left(\lambda_{k-L+1}\right)\right\}=\\ \arg\min\mathrm{KLD}\left(\left.q\left(x_{k-L:k}\right)q\left(Q_{k}\right)q\left(R_{k}\right)q\left(\xi_{k-L+1}\right)q\left(\lambda_{k-L+1}\right)\right\|p\left(\left.\Psi_{k}\right|z_{1:k}\right)\right) \end{array}$$

The optimal solution can be formulated as

(21) $$\log q\left(\theta \right)={Ε}_{\psi_{k}^{\left(-\theta \right)}}\left[\log p\left(\Psi_{k}|z_{1:k}\right)\right]+c_{\theta }$$

where θ denotes any element in set $\Psi_{k}$; $\Psi_{k}^{\left(-\theta \right)}$ represents all elements within set $\Psi_{k}$ except for θ; $c_{\theta }$ is a constant independent of the variable θ. The posterior joint PDF $p\left(\Psi_{k}|z_{1:k}\right)$ is written as follows:

(22) $$\begin{array}{l} p\left(\Psi_{k}|z_{1:k}\right)=\prod_{j=L+1}^{k}\left[\begin{array}{c} N\left(x_{j};F_{j}x_{j-1},Q_{j}/\xi_{j}\right)\times G\left(\xi_{j};\omega /2,\omega /2\right)\\ \times N\left(z_{j};H_{j}x_{j},R_{j}/\lambda_{j}\right)\times G\left(\lambda_{j};v/2,v/2\right) \end{array}\right]\\ \;\;\;\;\;\times \mathrm{IW}\left(Q_{k};{\hat{t}}_{k|k-L},{\hat{T}}_{k|k-L}\right)\times \mathrm{IW}\left(R_{k};{\hat{u}}_{k|k-L},{\hat{U}}_{k|k-L}\right)\\ \;\;\;\;\;\times N\left(x_{k-L};{\hat{x}}_{x-L|x-L},P_{x-L|x-L}\right)\times p\left(z_{1:k-L+1}\right) \end{array}$$

It can be seen from the equation that these components are mutually coupled. Therefore, a fixed-point iteration method is used to obtain their approximate solutions. This approach involves solving one component iteratively while keeping the other components fixed during each computation. $q\left(x_{k-L:k}\right)$ can be approximated by

(23) $$q^{\left(i+1\right)}\left(x_{x-L:k}\right)\propto N\left(x_{k-L:k};{\hat{x}}_{k-L:k-L},P_{k-L:k-L}\right)\times \prod_{j=k-L+1}^{k}\left[N\left(x_{j};F_{j}x_{j-1},{\tilde{Q}}_{j|k}^{\left(i\right)}\right)N\left(z_{j};H_{j}x_{j},{\tilde{R}}_{j|k}^{\left(i\right)}\right)\right]$$

where ${\hat{x}}_{k-L:k-L}$ and $P_{k-L:k-L}$ represent the state smoothing estimation of the slide window and PECM.; measurement noise covariance matrices are given as

(24) $${\tilde{Q}}_{\left.j\right|k}^{\left(i\right)}={\left\{{Ε}^{\left(i\right)}\left[Q_{k}^{-1}\right]\right\}}^{-1}/{Ε}^{\left(i\right)}{\left[\xi_{j}\right]}^{-1}$$

(25) $${\tilde{R}}_{\left.j\right|k}^{\left(i\right)}={\left\{{Ε}^{\left(i\right)}\left[R_{k}^{-1}\right]\right\}}^{-1}/{Ε}^{\left(i\right)}{\left[\lambda_{j}\right]}^{-1}$$

The iterative posterior distribution of the noise covariance matrix can be approximated as an inverse Wishart distribution:

(26) $$q^{\left(i+1\right)}\left(Q_{k}\right)=\mathrm{IW}\left(Q_{k};{\hat{t}}_{k|k}^{\left(i+1\right)},{\hat{T}}_{k|k}^{\left(i+1\right)}\right)$$

(27) $$q^{\left(i+1\right)}\left(R_{k}\right)=\mathrm{IW}\left(R_{k};{\hat{u}}_{k|k}^{\left(i+1\right)},{\hat{U}}_{k|k}^{\left(i+1\right)}\right)$$

where the parameters are updated by

(28) $${\hat{t}}_{k|k}^{\left(i+1\right)}={\hat{t}}_{k|k-L}+L$$

(29) $${\hat{T}}_{k|k}^{\left(i+1\right)}={\hat{T}}_{k|k-L}+\sum_{j=k-L+1}^{k}A_{j}^{\left(i+1\right)}{Ε}^{\left(i\right)}\left[\xi_{j}\right]$$

(30) $${\hat{u}}_{k|k}^{\left(i+1\right)}={\hat{u}}_{k|k-L}+L$$

(31) $${\hat{U}}_{k|k}^{\left(i+1\right)}={\hat{U}}_{k|k-L}+\sum_{j=k-L+1}^{k}B_{j}^{\left(i+1\right)}{Ε}^{\left(i\right)}\left[\lambda_{j}\right]$$

where the auxiliary matrices $A_{j}^{\left(i+1\right)}$ and $B_{j}^{\left(i+1\right)}$ are given by

(32) $$A_{j}=Ε\left[\left(x_{j}-F_{j}x_{j-1}\right){\left(x_{j}-F_{j}x_{j-1}\right)}^{T}\right]$$

(33) $$B_{j}=Ε\left[\left(z_{j}-H_{j}x_{j}\right){\left(z_{j}-H_{j}x_{j}\right)}^{T}\right]$$

The auxiliary matrix can be derived using expectation computation as

(34) $$A_{j}^{\left(i+1\right)}=P_{j|k}^{\left(i+1\right)}-F_{j}P_{j-1,j|k}^{\left(i+1\right)}-{\left[P_{j-1,j|k}^{\left(i+1\right)}\right]}^{T}F_{j}^{T}+F_{j}P_{j-1,j|k}^{\left(i+1\right)}F_{j}^{T}+\left({\hat{x}}_{j|k}^{\left(i+1\right)}-F_{j}{\hat{x}}_{j-1|k}^{\left(i+1\right)}\right){\left({\hat{x}}_{j|k}^{\left(i+1\right)}-F_{j}{\hat{x}}_{j-1|k}^{\left(i+1\right)}\right)}^{T}$$

(35) $$B_{j}^{\left(i+1\right)}=H_{j}P_{\left.j\right|k}^{\left(i+1\right)}H_{j}^{T}+\left(z_{j}-H_{j}x_{j|k}^{\left(i+1\right)}\right){\left(z_{j}-H_{j}x_{j|k}^{\left(i+1\right)}\right)}^{T}$$

where ${\hat{x}}_{j|k}^{\left(i+1\right)}$ and $P_{j|k}^{\left(i+1\right)}$ represent the smoothing estimate and the corresponding error covariance matrix at time j in the (*i* + 1)th iteration, and $P_{j-1,j|k}^{\left(i+1\right)}$ represents the smoothing errors cross-covariance matrix at times j−1 and j calculated as

(36) $$P_{j-1,j|k}^{\left(i+1\right)}=G_{j-1}^{\left(i+1\right)}P_{j|k}^{\left(i+1\right)}$$

where $G_{j-1}^{\left(i+1\right)}$ is the smoothing gain at time j−1.

The iterative posterior distributions of the auxiliary random variables are updated as the following Gamma distributions:

(37) $$q^{\left(i+1\right)}\left(\xi_{j}\right)=G\left(\xi_{j};{\hat{a}}_{j|k}^{\left(i+1\right)},{\hat{b}}_{j|k}^{\left(i+1\right)}\right)$$

(38) $$q^{\left(i+1\right)}\left(\lambda_{j}\right)=G\left(\lambda_{j};{\hat{c}}_{j|k}^{\left(i+1\right)},{\hat{d}}_{j|k}^{\left(i+1\right)}\right)$$

where the shape parameters and rate parameters are calculated as

(39) $${\hat{a}}_{j|k}^{\left(i+1\right)}=0.5\left(\omega +n\right)$$

(40) $${\hat{b}}_{j|k}^{\left(i+1\right)}=0.5\left[\omega +tr\left(A_{j}^{\left(i+1\right)}{Ε}^{\left(i\right)}\left[Q_{k}^{-1}\right]\right)\right]$$

(41) $${\hat{c}}_{j|k}^{\left(i+1\right)}=0.5\left(\nu +m\right)$$

(42) $${\hat{d}}_{j|k}^{\left(i+1\right)}=0.5\left[\nu +tr\left(B_{j}^{\left(i+1\right)}{Ε}^{\left(i\right)}\left[R_{k}^{-1}\right]\right)\right]$$

The expectations are computed as

(43) $${Ε}^{\left(i\right)}\left[Q_{k}^{-1}\right]={\hat{t}}_{k|k}^{\left(i\right)}/{\hat{T}}_{k|k}^{\left(i\right)}$$

(44) $${Ε}^{\left(i\right)}\left[R_{k}^{-1}\right]={\hat{u}}_{k|k}^{\left(i\right)}/{\hat{U}}_{k|k}^{\left(i\right)}$$

(45) $${Ε}^{\left(i\right)}\left[\xi_{k}\right]={\hat{a}}_{j|k}^{\left(i\right)}/{\hat{b}}_{j|k}^{\left(i\right)}$$

(46) $${Ε}^{\left(i\right)}\left[\lambda_{k}\right]={\hat{c}}_{j|k}^{\left(i\right)}/{\hat{d}}_{j|k}^{\left(i\right)}$$

### 3.3. Construction of Multiple Fading Factors

When the PNCM exhibits time-varying statistical properties, conventional Kalman filters often suffer from slow convergence and may yield inaccurate estimates. Accordingly, a single fading factor is introduced to adjust the PECM, aiming to improve filtering accuracy and reduce estimation errors. In practice, the estimation performance of each component in the PECM differs, and uncertainty in the system state and noise covariances affects the PECM diagonal elements differently, thus limiting the application of single fading factors [25].

To address this limitation and reduce the impact of PECM on estimation accuracy, a multi-fading factor approach is proposed. This method enhances the adjustment capability of individual filtering channels, thereby improving the overall performance of the algorithm. Multiple fading factors are given in the following matrix form:

(47) $$\Delta_{k}=diag\left(\lambda_{1,k},\lambda_{2,k},\ldots ,\lambda_{k,k}\right)$$

(48) $$\lambda_{i,k}=\gamma_{i}\alpha_{k},i=1,\ldots ,n$$

where $\alpha_{k}$ is the scalar fading factor at time k; $\gamma_{k}$ represents the weight of the fading factor corresponding to the *i*th state component.

According to the strong tracking theory, $\alpha_{k}$ is described as follows:

(49) $$\alpha_{k}=\left\{\begin{array}{c} \tilde{\alpha },\tilde{\alpha }>1\\ 1,\tilde{\alpha }\leq 1 \end{array}\begin{array}{c} , \end{array}\tilde{\alpha }=\right.tr\left[N_{k}\right]/\sum_{i=1}^{l}\rho_{i}M_{k}^{ii}$$

(50) $$N_{k}=C_{k}-H_{k}{\hat{Q}}_{k-1}H_{k}^{T}-\beta {\hat{R}}_{k}$$

(51) $$M_{k}=H_{k}F_{k-1}P_{k-1|k-1}F_{k-1}^{T}H_{k}^{T}$$

where $C_{k}$ denotes the covariance matrix of the output residual vector; ${\hat{Q}}_{k-1}$ and ${\hat{R}}_{k}$ are the estimated PNCM and MNCM by the inverse Wishart distribution, respectively. In general, $C_{k}$ is obtained by a windowing method [26]. In this paper, an exponential weighting method based on fading memory is adopted. The recursive formula of $C_{k}$ at time k is defined as

(52) $$C_{k}=\left\{\begin{array}{c} e_{1}e_{1}^{T}\\ \left(1-\delta \right)e_{k}e_{k}^{T}+\left(\delta -\delta^{k}\right)C_{k-1}/\left(1-\delta^{k}\right) \end{array}\begin{array}{c} ,k=1\\ ,k>1 \end{array}\right.$$

where $e_{k}=z_{k}-H_{k}{\hat{x}}_{k|k-1}$ represents the state residual vector; δ is the forgetting factor, which enhances the tracking performance of the algorithm by amplifying the influence of the residual sequence. The PECM $P_{k|k-1}$ is primarily regulated by the multiple fading factors:

(53) $${\hat{P}}_{k|k-1}=\Delta_{k}F_{k-1}P_{k-1|k-1}F_{k-1}^{T}+{\hat{Q}}_{k-1}$$

The proposed method operates sequentially via time update, multiple fading factor calculation, measurement update, state smoothing, and data saving. The corresponding pseudocode is formally presented in Algorithm 1.
**Algorithm 1** The proposed SWVKF-ST**Inputs:** $\left\{{\hat{x}}_{j|j},P_{j|j},{\hat{t}}_{j|j},{\hat{T}}_{j|j},{\hat{u}}_{j|j},{\hat{U}}_{j|j},z_{j}|k-L\leq j\leq k-1\right\}$, $\left\{{\hat{\xi }}_{j|k-1},{\hat{\lambda }}_{j|k-1}|k-L+1\leq j\leq k-1\right\}$, $Q_{k}$, $R_{k}$, $F_{k}$, $H_{k}$, ω, ν, ρ, ε, δ, $N_{m}$.**Initialization:****For** j=k−L+1:k−1 $\xi_{j}={\hat{\xi }}_{j|k-1}$, $\lambda_{j}={\hat{\lambda }}_{j|k-1}$**End for**Calculate DOF parameters and inverse-scale matrices using (24)–(25)**Iteration:****For** $i=0:N_{m}-1$ 
${\hat{x}}_{j|j-1}^{\left(i+1\right)}=F_{j}{\hat{x}}_{j-1|j-1}^{\left(i+1\right)}$
 Calculate the scale matrices ${\tilde{Q}}_{j}^{\left(i\right)}$ and ${\tilde{R}}_{j}^{\left(i\right)}$ using (31)–(32) Obtain the multiple fading factors $\Delta_{j}$ by (11)–(15) 
${\hat{P}}_{j|j-1}^{\left(i+1\right)}=\Delta_{j}F_{j}P_{j-1|j-1}^{\left(i+1\right)}F_{j}^{T}+{\tilde{Q}}_{j}^{\left(i\right)}$
 
$K_{j}^{\left(i+1\right)}=P_{j|j-1}^{\left(i+1\right)}H_{j}^{T}\left(H_{j}P_{j|j-1}^{\left(i+1\right)}H_{j}^{T}+{\tilde{R}}_{j}^{\left(i\right)}\right)$
 
${\hat{x}}_{j|j}^{\left(i+1\right)}={\hat{x}}_{j|j-1}^{\left(i+1\right)}+K_{j}^{\left(i+1\right)}\left(z_{j}-H_{j}{\hat{x}}_{j|j-1}^{\left(i+1\right)}\right)$
 
$P_{j|j}^{\left(i+1\right)}=P_{j|j-1}^{\left(i+1\right)}-K_{j}^{\left(i+1\right)}H_{j}P_{j|j-1}^{\left(i+1\right)}$
**End for****For** $j=k:\left(-1\right):k-L+1$ 
$G_{j-1}^{\left(i+1\right)}=P_{j-1|j-1}^{\left(i+1\right)}F_{j}^{T}{\left[P_{j|j-1}^{\left(i+1\right)}\right]}^{-1}$
 
${\hat{x}}_{j-1|k}^{\left(i+1\right)}={\hat{x}}_{j-1|j-1}^{\left(i+1\right)}+G_{j-1}^{\left(i+1\right)}\left({\hat{x}}_{j|k}^{\left(i+1\right)}-F_{j}{\hat{x}}_{j-1|j-1}^{\left(i+1\right)}\right)$
 
$P_{j-1|k}^{\left(i+1\right)}=P_{j-1|j-1}^{\left(i+1\right)}+G_{j-1}^{\left(i+1\right)}\left(P_{j|k}^{\left(i+1\right)}-P_{j|j-1}^{\left(i+1\right)}\right){\left[G_{j-1}^{\left(i+1\right)}\right]}^{T}$
 Calculate $A_{j}^{\left(i+1\right)}$ and $B_{j}^{\left(i+1\right)}$ as (39)–(40) Calculate the shape parameters and rate parameters using (44)–(47) Calculate the expectations as (48)–(51) If $\left\|{\hat{x}}_{k|k}^{\left(i+1\right)}-{\hat{x}}_{k|k}^{\left(i\right)}\right\|/\left\|{\hat{x}}_{k|k}^{\left(i\right)}\right\|\leq \varepsilon$, the iteration is terminated**End for**${\hat{x}}_{k|k}={\hat{x}}_{k|k}^{\left(i\right)}$, $P_{k|k}=P_{k|k}^{\left(i+1\right)}$${\hat{t}}_{k|k}={\hat{t}}_{k|k}^{\left(i+1\right)}$, ${\hat{T}}_{k|k}={\hat{T}}_{k|k}^{\left(i+1\right)}$${\hat{u}}_{k|k}={\hat{u}}_{k|k}^{\left(i+1\right)}$, ${\hat{U}}_{k|k}={\hat{U}}_{k|k}^{\left(i+1\right)}$**Outputs**:$\left\{{\hat{x}}_{j|j},P_{j|j},{\hat{t}}_{j|j},{\hat{T}}_{j|j},{\hat{u}}_{j|j},{\hat{U}}_{j|j}|k-L\leq j\leq k-1\right\}$, $Q_{k}$, $R_{k}$, $C_{k}$. 

## 4. Simulations and Experiment

To evaluate the navigation performance of the proposed method, both simulation and lake experiments are designed. The process starts with tuning and analyzing algorithm parameters, and then proceeds to comparative studies against existing filtering-based localization methods. The effectiveness and robustness of the method are further validated through real-world experiments.

### 4.1. Simulations

Similar to existing research [27], this paper adopts the continuous white noise acceleration model in 2D Cartesian coordinates to the performance of the proposed algorithm. In the simulations of maneuvering target tracking, we define the state vector as $X_{k}={\left[x_{k},y_{k},{\dot{x}}_{k},{\dot{y}}_{k}\right]}^{T}$, where $x_{k}$ and $y_{k}$ represent the abscissa and ordinate of the target and ${\dot{x}}_{k}$ and ${\dot{y}}_{k}$ denote the corresponding velocities. The simulation model is set up according to Equation (1), where the state transition matrix $F_{k}=\left[I_{2},\Delta tI_{2};0,I_{2}\right]$ and the system measurement observation matrix $H_{k}=\left[I_{2},0\right]$, where the parameter Δt=1s is the sampling interval and $I_{2}$ is the 2D identity matrix. The time-varying models of true PNCM and MNCM are given by [28]

(54) $$Q_{k}=\left[6.5+0.5\cos\left(\frac{10\pi k}{T}\right)\right]\times q\left[\frac{\Delta t^{3}}{3}I_{2},\frac{\Delta t^{2}}{2}I_{2};\frac{\Delta t^{3}}{3}I_{2},\Delta tI_{2}\right]$$

(55) $$R_{k}=\left[0.1+0.05\cos\left(\pi k/T\right)\right]\times r\left[1,0.5;0.5,1\right]$$

where T=1000 s is the total time of simulation test; $q=1{\;m}^{2}/s^{3}$ and $r={100\;m}^{2}$ are scale factors. Moreover, the nominal covariance matrices are, respectively, selected as ${\tilde{Q}}_{k}=\alpha I_{4}$ and ${\tilde{R}}_{k}=\eta I_{2}$, where α and η are prior confidence parameters for adjusting the initial noise covariances. To further simulate underwater environmental conditions, the noise affecting the measurement process is generated based on the following factors:

(56) $$\left\{\begin{array}{l} v_{k}∼\left\{\begin{array}{l} N\left(0,{\tilde{R}}_{k}\right),\;\;\;p=0.90\\ N\left(0,100{\tilde{R}}_{k}\right),\;\;\;\;p=0.10 \end{array}\right.\\ w_{k}∼\left\{\begin{array}{l} N\left(0,{\tilde{Q}}_{k}\right),\;\;p=0.90\\ N\left(0,100{\tilde{Q}}_{k}\right),\;\;\;p=0.10 \end{array}\right. \end{array}\right.$$

To verify the superiority of the proposed algorithm, the SWVKF-ST proposed algorithm is compared to the existing filtering algorithms including the nominal KF (NKF) based on nominal PNCM and MNCM, the true KF (TKF) based on true PNCM and MNCM, SSMKF, RSTKF, and SWRKF algorithms.

In the simulation test, the configurations of other filters sharing parameters with the proposed algorithm are as follows: the prior confidence parameters α=1 and η=100, the window lengths L=10, the number of iterations N=15, and the changing factor $\rho =1-e^{-4}$. In addition, since the DOF parameters in Student’s *t*-distribution significantly influence algorithm performance, it is important to explicitly specify the DOF values used in different algorithms. On the one hand, large DOF parameters weaken the algorithm’s ability to suppress abnormal noise; on the other hand, excessively small DOF parameters can make the algorithm overly sensitive, leading to estimation errors. Hence, referring to the exiting research [17], the DOF parameters of the proposed SWVKF-ST, RSTKF, SWRKF algorithm for Student’s *t* noise are set to ω=ν=5, respectively. Moreover, the initial state is set as $x_{0}={\left[0,0;10,10\right]}^{T}$, and the corresponding error covariance matrix is selected as $P_{0}=diag\left(\left[10000,10000;100,100\right]\right)$.

To evaluate the system state estimation accuracy, two assessment indexes for the position and velocity including root mean square errors (RMSEs) and average RMSEs (ARMSEs) can be used as follows:

(57) $$\left\{\begin{array}{l} \mathrm{RMSE}≜\sqrt{\frac{1}{N}\sum_{s=1}^{N}\left({\left(x_{k}^{s}-{\hat{x}}_{k}^{s}\right)}^{2}+{\left(y_{k}^{s}-{\hat{y}}_{k}^{s}\right)}^{2}\right)}\\ \mathrm{ARMSE}≜\frac{1}{T}\sum_{k=1}^{T}\sqrt{\frac{1}{N}\sum_{s=1}^{N}\left({\left(x_{k}^{s}-{\hat{x}}_{k}^{s}\right)}^{2}+{\left(y_{k}^{s}-{\hat{y}}_{k}^{s}\right)}^{2}\right)} \end{array}\right.$$

where $\left(x_{k}^{s},y_{k}^{s}\right)$ and $\left({\hat{x}}_{k}^{s},{\hat{y}}_{k}^{s}\right)$ are, respectively, the true and estimated positions or velocities in the *s*th Monte-Carlo experiment run, and N=1000 represents the total number of Monte-Carlo runs. Furthermore, to assess the state estimation’s consistency, the normalized estimate error squared (NEES) is defined by

(58) $$\mathrm{NEES}=\frac{1}{N}\sum_{s=1}^{N}{\left(x_{k}^{s}-{\hat{x}}_{k}^{s}\right)}^{T}{\left(P_{k|k}^{s}\right)}^{-1}\left(x_{k}^{s}-{\hat{x}}_{k}^{s}\right)$$

where $x_{k}^{s}$, ${\hat{x}}_{k}^{s}$, and $P_{k|k}^{s}$ represent the true and estimated states and the corresponding error covariance.

**Figure 3:**
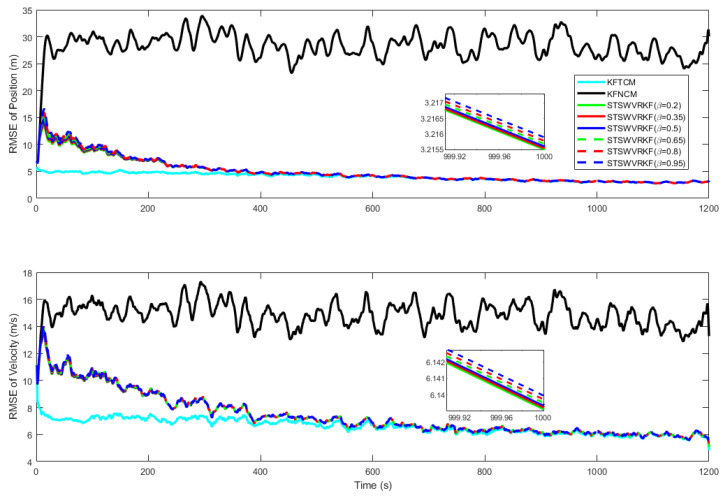
RMSEs of the position and velocity at β=0.2:0.15:0.95.

Figure 3 shows the RMSEs of position and velocity at β=0.2,0.35,0.5,0.65,0.8,0.95, with a zoomed-in view at t=1000s. It can be seen from Figure 3 that the RMSE from SWVKF-ST is significantly smaller than that from NKF. As parameter β decreases, the RMSE shows a decreasing trend and gradually approaches that of TKF. When β=0.2, it is closest to TKF.

**Figure 4:**
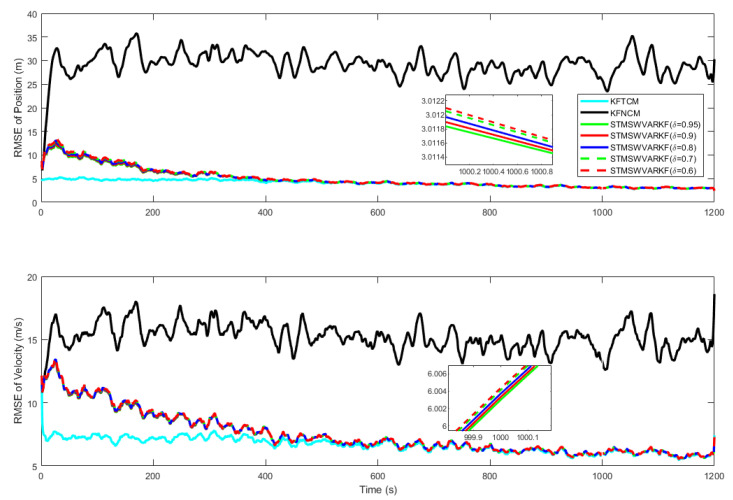
RMSEs of position and velocity at δ=0.6,0.7,0.8,0.9,0.95.

In order to analyze the influence of forgetting factor δ on the filtering performance, TKF, NKF, and SWVKF-ST with different δ are tested separately, where β=0.2. Figure 4 shows the RMSEs of position and velocity at δ=0.6,0.7,0.8,0.9,0.95, with a zoomed-in view at t=1000 s. As can be seen from Figure 4, both in terms of position and velocity, the proposed SWVKF-ST has higher estimation accuracy compared to NKF. When δ increases, the RMSEs of position and velocity decrease, and filtering convergence speed slows down to different degrees. It can be observed that regardless of the parameter values, the proposed algorithm consistently outperforms the NKF in terms of estimation accuracy.

**Figure 5:**
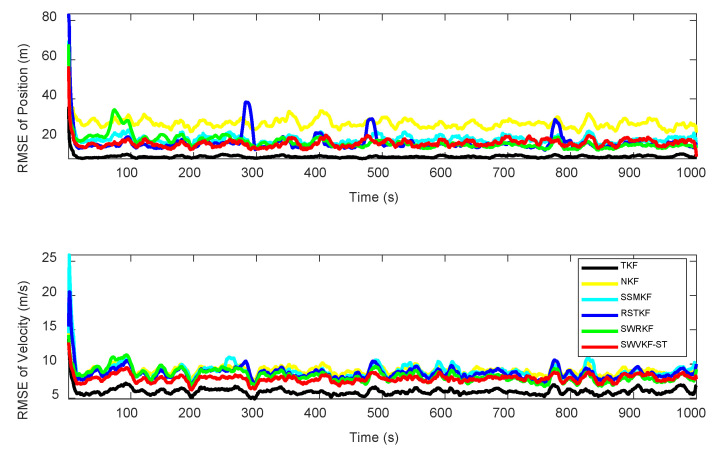
RMSEs of position and velocity for different algorithms.

To facilitate a fair comparison between the proposed method and other filtering algorithms, the weakening factor and forgetting factor are set as β=0.2 and δ=0.95, respectively. Figure 5 and Table 1 show the RMSEs and ARMSEs of the position and velocity for different algorithms. From Figure 5, the convergence speed and estimation accuracy for the position and velocity with SWVKF-ST are closest to TKF. In addition, the proposed algorithm demonstrates enhanced robustness to outliers, enabling it to achieve superior estimation accuracy and reliability in complex and noisy environments. It can be observed from Table 1 that the ARMSEs of SWVKF-ST are the smallest among the five filtering algorithms.

**Figure 6:**
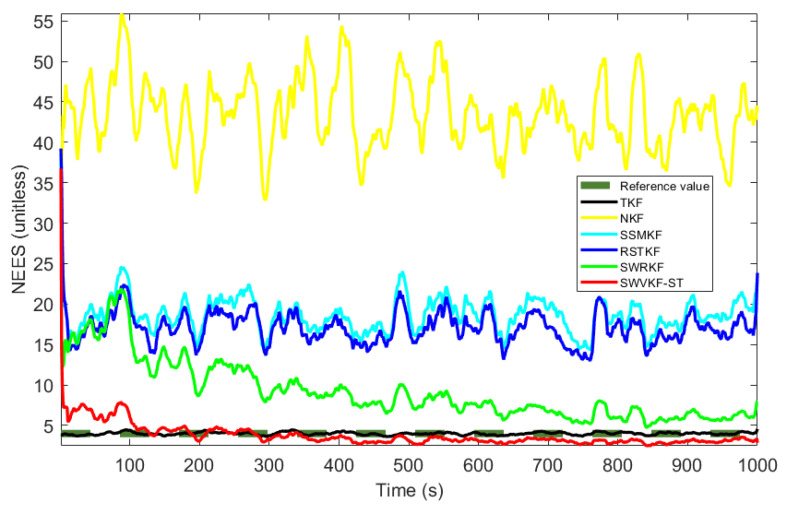
NEESs of different algorithms.

Figure 6 and Table 2 illustrate the NEESs and ANEESs of the proposed SWVKF-ST and other filtering algorithms. As shown in Figure 6, we can see that the proposed SWVKF-ST consistently exhibits the lowest NEES values, even outperforming TKF, which suggests that it achieves the best consistency among all evaluated filters. Table 2 lists the ANEESs and single step run time of different algorithms. As shown in Table 2, the proposed method achieves significantly improved estimation accuracy despite a slight increase in computational cost. In summary, compared to existing filters, the proposed SWVKF-ST algorithm demonstrates superior effectiveness in mitigating the impact of outliers and provides higher estimation accuracy for the state vector.

### 4.2. Surface Vessel Test

**Figure 7:**
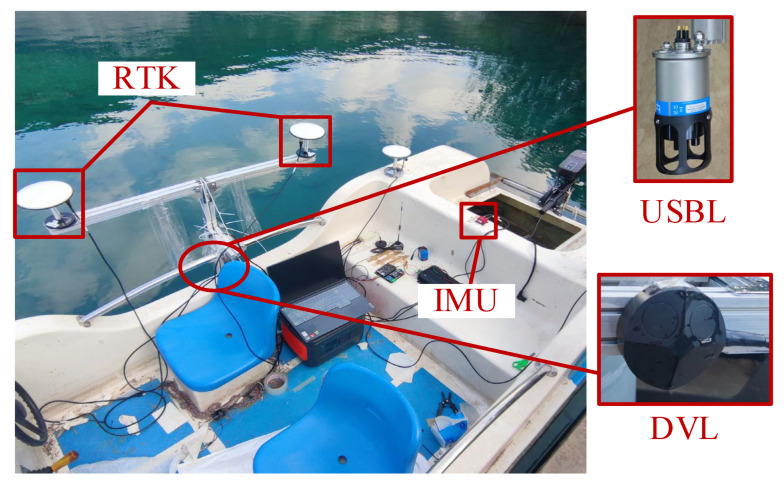
Experimental platform for fusion positioning algorithm test.

Sensor measurement errors are typically more significant in the horizontal plane, while vertical errors are generally corrected using depth sensors such as pressure gauges. Therefore, the focus of this study is primarily on horizontal-plane errors. DVL can effectively collect underwater velocity information. Moreover, since underwater vehicles typically operate at low and relatively constant speeds, the impact of IMU performance limitations is relatively minor. Due to environmental interference and multipath effects, the USBL system often produces unreliable position data. To obtain accurate reference values, we adopt an RTK system as the reference standard. As a result, surface experiments are conducted instead of underwater experiments, providing a controlled and reliable validation environment for the proposed algorithm. Two-dimensional navigation and localization experiments are thus carried out to evaluate the algorithm’s robustness and effectiveness under simulated underwater conditions with realistic sensor limitations. To further assess the performance of the proposed SWVKF-ST algorithm in complex underwater environments, a comprehensive set of integrated navigation experiments was conducted at a test lake in Xuyi City, Jiangsu Province. The configuration of the experimental platform is depicted in Figure 7. This platform incorporates multiple sensors, including RTK, IMU, DVL, and USBL, to enable the acquisition of diverse observational datasets. Table 3 shows the specific parameters of IMU, DVL, and RTK.

The test site presented significant challenges for navigation due to environmental factors. First, multipath effects are frequently observed in acoustic signals, where reflections from the water surface, bottom, and nearby terrain introduce delayed and distorted measurements. These effects cause deviations in sensor readings, particularly in the DVL systems, leading to outliers. Second, signal attenuation is notable in this environment due to factors such as suspended particulates and thermocline-induced acoustic scattering, which weaken the strength and integrity of acoustic signals. In addition, the presence of mountainous terrain around the lake interferes with the reception of electromagnetic signals, resulting in signal loss and positioning uncertainty. Together, these environmental factors create a highly realistic and harsh setting for evaluating the proposed algorithm’s robustness against non-Gaussian noise and sensor outlier.

**Figure 8:**
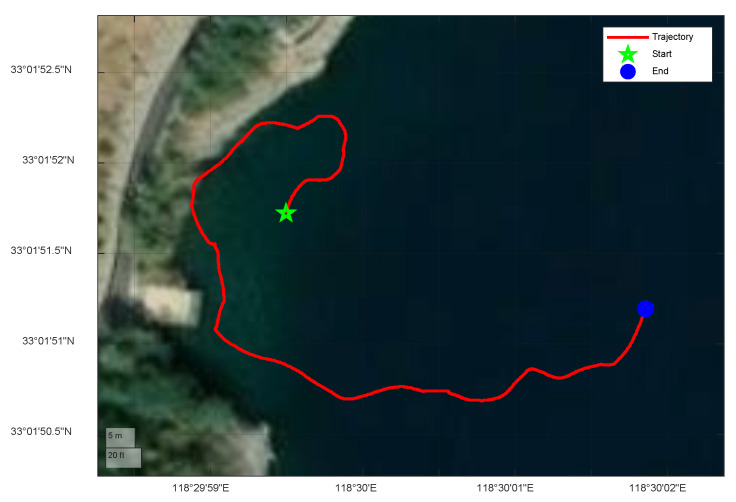
Surface vessel trajectory.

**Figure 9:**
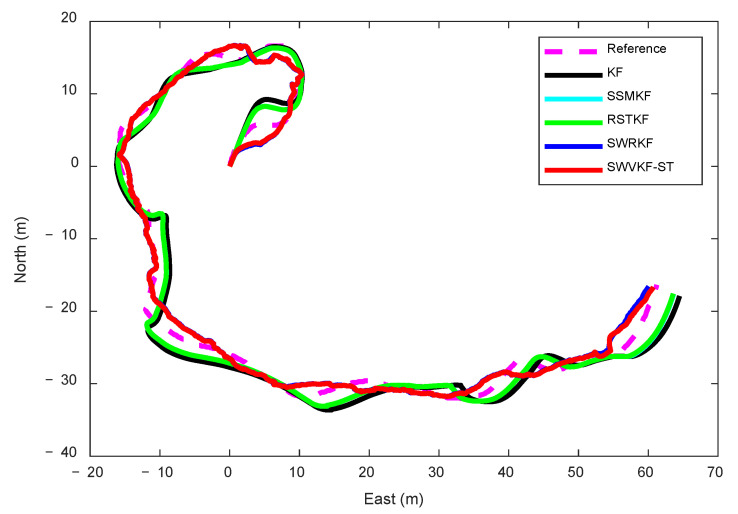
Target trajectory in different algorithms.

The lake experiment lasts for 230 s, and the actual surface vessel trajectory of the lake experiment in shown in Figure 8. The trajectories estimated by the proposed algorithm and comparative methods are shown in Figure 9. The results clearly demonstrate that the trajectory estimated by the proposed SWVKF-ST algorithm aligns significantly closer to the reference trajectory than those estimated by conventional methods, highlighting its superior accuracy in path estimation.

**Figure 10:**
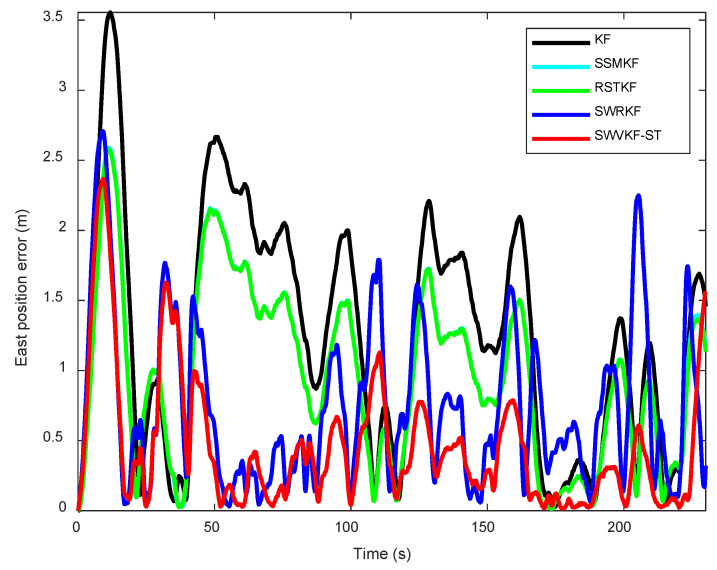
East position errors of different algorithms.

**Figure 11:**
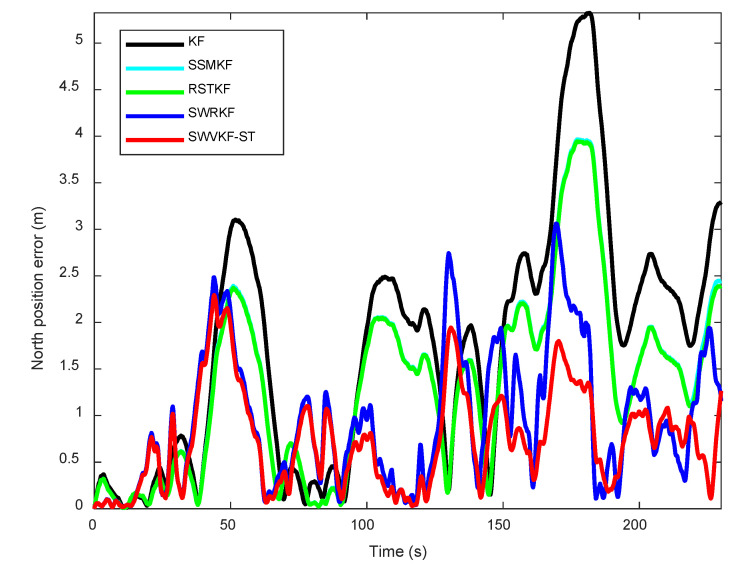
North position errors of different algorithms.

To further investigate the performance of different algorithms, the dynamic estimation errors in the northward and eastward directions were analyzed in detail. Figure 10 and Figure 11 illustrate the proposed method exhibits smaller estimation errors in both the east and north directions compared to other algorithms. Moreover, to facilitate a comprehensive quantitative evaluation, the errors for positioning estimates in the northward and eastward directions were tabulated, offering a comparative summary of the algorithms’ performances. As observed from Table 4, the proposed SWVKF-ST algorithm consistently achieves the smallest estimation errors among all methods. Compared to SWRKF, it demonstrates slight improvements in positioning accuracy for both the eastward and northward directions. This demonstrates its outstanding capability in accurately estimating state trajectories while maintaining robustness against environmental disturbances and measurement outliers.

## 5. Conclusions

To address the problem of heavy-tailed noise affecting positioning accuracy in multi-sensor fusion, this paper proposes a robust SWVKF-ST algorithm. From a practical perspective, the proposed method employs a sliding window to utilize these measurements over a period of time, thereby enhancing outlier detection capability and improving posterior accuracy. Additionally, this method introduces heavy-tailed noise modeling into the KF framework via variational inference, significantly enhancing the robustness and reliability of underwater localization in the presence of outliers and noise interference. Furthermore, the incorporation of multiple fading factors based on the strong tracking principle enhances adaptability to time-varying noise. From a practical perspective, it is demonstrated through simulation and experimental results that the proposed method achieves higher estimation accuracy and reliability than the compared methods in challenging underwater scenarios. However, the increased computational complexity due to the use of sliding windows and variational inference may hinder real-time implementation on resource-constrained platforms. Additionally, the current approach assumes prior knowledge of the noise distribution form, which may limit adaptability in scenarios with mixed noise characteristics. Future work will focus on further optimizing computational efficiency, exploring adaptive noise model selection, and validating the algorithm in more complex real-world environments.

## Figures and Tables

**Table 1:** ARMSEs of position and velocity for different algorithms.

Algorithms	ARMSE_pos_ (m)	ARMSE_vel_ (m/s)
TKF	10.4871	6.0097
NKF	27.4480	9.1783
SSMKF	19.3816	8.9235
RSTKF	17.3648	8.6984
SWRKF	17.7961	8.3188
SWVKF-ST	17.3249	7.8931

**Table 2:** ANEESs and single step run time of different algorithms.

Algorithms	ANEES	Times (ms)
TKF	3.9911	0.0078
NKF	43.5038	0.0078
SSMKF	18.6296	0.0656
RSTKF	17.0918	0.0982
SWRKF	9.2505	0.3865
SWVKF-ST	3.7192	0.4327

**Table 3:** Parameters of sensors in surface vessel test.

Sensor	Specifications
IMU	Type: Ellipes−E Frequency: 200 Hz Gyroscope random walk: $0.18{}^{\circ}/\sqrt{hr}$ Gyroscope in run bias instability: 7°/hr Accelerometer random walk: $57\;\mu g/\sqrt{\mathrm{hz}}$ Accelerometer in run bias instability: 14 μg
DVL	Type: DVL−A50 Frequency: 10 Hz Velocity resolution: 0.1 mm/s Long term accuracy: 0.1%
RTK	Type: BYNAV X1 Frequency: 1 Hz Positioning accuracy: 1.5 cm

**Table 4:** Position errors of different algorithms.

Algorithms	East Error	North Error
KF	1.2554	1.8327
SSMKF	0.9588	1.3414
RSTKF	0.9547	1.3300
SWRKF	0.7406	0.9560
STSWVRKF	0.4509	0.7414

## Data Availability

The data are available from the lead author upon reasonable request.
